# First-Principle Validation of Fourier’s Law: One-Dimensional Classical Inertial Heisenberg Model

**DOI:** 10.3390/e26010025

**Published:** 2023-12-25

**Authors:** Henrique Santos Lima, Constantino Tsallis, Fernando Dantas Nobre

**Affiliations:** 1 Centro Brasileiro de Pesquisas Físicas, Rua Xavier Sigaud 150, Rio de Janeiro 22290-180, RJ, Brazil; tsallis@cbpf.br (C.T.); fdnobre@cbpf.br (F.D.N.); 2 National Institute of Science and Technology for Complex Systems, Rua Xavier Sigaud 150, Rio de Janeiro 22290-180, RJ, Brazil; 3 Santa Fe Institute, 1399 Hyde Park Road, Santa Fe, NM 87501, USA; 4 Complexity Science Hub Vienna, Josefstädter Strasse 39, 1080 Vienna, Austria

**Keywords:** Fourier’s law, generalized entropies, non-equilibrium physics, stochastic processes

## Abstract

The thermal conductance of a one-dimensional classical inertial Heisenberg model of linear size *L* is computed, considering the first and last particles in thermal contact with heat baths at higher and lower temperatures, $T_{h}$ and $T_{l}$ ( $T_{h}>T_{l}$ ), respectively. These particles at the extremities of the chain are subjected to standard Langevin dynamics, whereas all remaining rotators ( i=2,⋯,L−1 ) interact by means of nearest-neighbor ferromagnetic couplings and evolve in time following their own equations of motion, being investigated numerically through molecular-dynamics numerical simulations. Fourier’s law for the heat flux is verified numerically, with the thermal conductivity becoming independent of the lattice size in the limit L→∞ , scaling with the temperature, as $\kappa \left(T\right)∼T^{-2.25}$ , where $T=(T_{h}+T_{l})/2$ . Moreover, the thermal conductance, σ(L,T)≡κ(T)/L , is well-fitted by a function, which is typical of nonextensive statistical mechanics, according to $\sigma \left(L,T\right)=A\exp_{q}\left(-Bx^{\eta }\right)$ , where *A* and *B* are constants, $x=L^{0.475}T$ , q=2.28±0.04 , and η=2.88±0.04 .

## 1. Introduction

Two centuries ago, Fourier proposed the law for heat conduction in a given macroscopic system, where the heat flux varies linearly with the temperature gradient, J∝−∇T [1]. For a simple one-dimensional system (e.g., a metallic bar along the $\hat{x}$ axis, $J=J\hat{x}$ ), the heat flux *J* (rate of heat per unit area) is given by

(1) $$J=-\kappa \;\frac{dT}{dx}\;,\;$$

where κ is known as thermal conductivity. In principle, κ may depend on the temperature, although most measurements are carried at room temperature, leading to values of κ for many materials (see, e.g., Ref. [2]). Usually, metals (like silver, copper, and gold) present large values of κ , and are considered good heat conductors, whereas poor heat conductors (such as air and glass fiber) are characterized by small thermal conductivities; typically, the ratio between the thermal conductivities of these two limiting cases may differ by a $10^{4}$ factor. In most cases, good thermal conductors are also good electrical conductors, and obey the Wiedemann–Franz law, which states that the ratio of their thermal and electrical conductivities follows a simple formula, being directly proportional to the temperature [3].

In recent years, numerous studies have been conducted to validate Fourier’s law in a wide variety of physical systems, both experimentally and theoretically. Particularly, investigations for which microscopic ingredients may be responsible for the property of heat conduction were carried out, and it has been verified that thermal conductivity may be generated by different types of particles (or quasi-particles). In the case of good electrical conductors, the most significant contribution to thermal conductivity comes from free electrons, whereas in electrical insulators, such contributions may arise from quasi-particles, like phonons and magnons, or even from defects. For instance, for antiferromagnetic electrical insulators, such as Sr_2_CuO_3_ and SrCuO_2_, which surprisingly behave as S=1/2 .

Heisenberg chains, magnons yield the most relevant contribution for the thermal conductivity, which can be fitted by a $1/T^{2}$ law, at high temperatures [4]. In these materials, the low-temperature regime presents ballistic-like heat conduction, increasing as the size of the system increases, while the high-temperature regime presents normal heat conduction [5,6,7].

Being a classical result, there is, in principle, no reason why Fourier’s law should generally apply to physical systems. This aspect has generated controversies in the literature, both in experimental and theoretical studies (for a comprehensive theoretical discussion, see, e.g., Ref. [8]). As a typical anomaly, the thermal conductivity κ (which should be an intensive quantity) appears, in many cases, to depend on the size of the system, e.g., on the total number of constituents, as it happens for chains of nonlinear oscillators, where κ increases with the total number of elements [9]. This anomaly is usually considered a failure of Fourier’s law. In addition, non-Fourier heat conduction can also emerge from the Maxwell–Cattaneo–Vernotte hyperbolic heat equation, which represents the relativistic version of the heat equation [10]. Recent advances in non-Fourier heat conduction can be found in the work by Benenti et al. [11].

Several experimental investigations have verified Fourier’s law in a diverse range of systems [4,12,13,14,15], including coal and rocks from coalfields [13], as well as two-dimensional materials [14,15]. On the other hand, some authors claim to have found anomalies [16], or even violations of this law for silicon nanowires [17], carbon nanotubes [18], and low-dimensional nanoscale systems [19]. Furthermore, a curious crossover, induced by disorder, was observed in quantum wires, where, by gradually increasing disorder, one goes from a low-disorder regime, where the law is apparently not valid, to another regime characterized by a uniform temperature gradient inside the wire, in agreement with Fourier’s law [20,21].

From the theoretical point of view, many authors have investigated Fourier’s law in a wide diversity of models [9,22,23,24,25,26,27,28,29,30,31,32,33,34,35,36,37,38,39,40,41,42,43,44,45,46], like a Lorentz gas [23], biological [30] and small quantum systems [29], chains of coupled harmonic [31] or anharmonic [9,28,34] oscillators, models characterized by long-range [38,46] or disordered [41] interactions, as well as systems of coupled classical rotators [42,43,44,45]. In the case of a coupled XY nearest-neighbor-interacting rotator chain [44], the temperature dependence of the thermal conductance was well-fitted by a *q*-Gaussian distribution,

(2) $$P_{q}\left(u\right)=P_{0}\exp_{q}\left(-\beta u^{2}\right)\;,\;$$

defined in terms of the *q*-exponential function,

(3) $$\exp_{q}\left(u\right)={\left[1+\left(1-q\right)u\right]}_{+}^{1/(1-q)}\;;\;\left(\exp_{1}\left(u\right)=\exp\left(u\right)\right)\;,\;$$

where $P_{0}\equiv P_{q}\left(0\right)$ and ${\left[y\right]}_{+}=y$ , for y>0 (zero otherwise). The distribution in Equation (2) is very common in the context of nonextensive statistical mechanics [47], since it appears from the extremization of the generalized entropy, known as $S_{q}$ , characterized by a real index *q* [48],

(4) $$S_{q}=k\sum_{i=1}^{W}p_{i}\left(\ln_{q}\frac{1}{p_{i}}\right)\;,$$

where we introduced the *q*-logarithm definition,

(5) $$\ln_{q}u=\frac{u^{1-q}-1}{1-q}\;;\;\left(\ln_{1}u=\ln u\right)\;.\;$$

Therefore, one recovers Boltzmann–Gibbs (BG) entropy,

(6) $$S_{\mathrm{BG}}=-k\sum_{i=1}^{W}p_{i}\ln p_{i}\;,\;$$

as $\lim_{q\to 1}S_{q}=S_{\mathrm{BG}}$ , whereas in the microcanonical ensemble, where all microstates present equal probability, $p_{i}=1/W$ , Equation (4) becomes,

(7) $$S_{q}=k\ln_{q}W\;.\;$$

Above, the *q*-exponential function in Equation (3) appears precisely as the inverse function of the *q*-logarithm of Equation (5), i.e., $\exp_{q}\left(\ln_{q}u\right)=\ln_{q}\left(\exp_{q}\left(u\right)\right)=u$ .

Since the introduction of the entropy $S_{q}$ in Equation (4), a large amount of works appeared in the literature, defining generalized functions and distributions (see, e.g., Ref. [47]). In particular, a recent study based on superstatistics has found a stretched *q*-exponential probability distribution [49],

(8) $$P_{q}\left(u\right)=P_{0}\exp_{q}{\left(-\beta |u\right|}^{\eta })\;\left(0<\eta \leq 1\right),\;$$

as well as its associated entropic form.

As already mentioned, the latest advances in experimental techniques made it possible to investigate thermal and transport properties and, hence, Fourier’s law, in low-dimensional (or even finite-size) systems, like two-dimensional materials [14,15], silicon nanowires [17], carbon nanotubes [18], and low-dimensional nanoscale systems [19]. These measurements motivate computational studies in finite-size systems of particles that present their own equations of motion, e.g., systems of interacting classical rotators, whose dynamics may be followed through the direct integration of their equations of motion. In this way, one may validate (or not) Fourier’s law, by computing the temperature and size dependence of the thermal conductance. A recent analysis of a system of coupled nearest-neighbor-interacting classical XY rotators [45], on *d*-dimensional lattices ( d=1,2,3 ) of linear size *L*, has shown that, for a wider range of temperatures, the temperature dependence of the thermal conductance was better fitted by a more general ansatz than the *q*-Gaussian distribution of Equation (2). In fact, Fourier’s law was validated in Ref. [45] by fitting the thermal conductance in terms of the functional form of Equation (8), with values of η(d)>2 .

In the present work, we analyze the thermal conductance of a one-dimensional classical inertial Heisenberg model of linear size *L*, considering the first and last particles in thermal contact with heat baths at temperatures $T_{h}$ and $T_{l}$ ( $T_{h}>T_{l}$ ), respectively. All remaining rotators ( i=2,⋯,L−1 ) interact by means of nearest-neighbor ferromagnetic couplings and evolve in time through molecular-dynamics numerical simulations. For this classical model, we specifically concentrate on the high-temperature limit, where there is no need for a spin wave approach, such as the Holstein–Primakoff quantum transformations. Our numerical data validate Fourier’s law, and similar to those of Ref. [45], the thermal conductance is well-fitted by the functional form of Equation (8). The present results suggest that this form should apply in general for the thermal conductance of nearest-neighbor-interacting systems of classical rotators. In the Section 2, we define the model and the numerical procedure; in Section 3, we present and discuss our results; in Section 4, we present our conclusions.

## 2. Materials and Methods

The one-dimensional classical inertial Heisenberg model, for a system of *L*-interacting rotators, is defined by the Hamiltonian,

(9) $$\begin{array}{c} H=\frac{1}{2}\sum_{i=1}^{L}\ell_{i}^{2}+\frac{1}{2}\sum_{\langle ij\rangle }\left(1-S_{i}\cdot S_{j}\right)\;, \end{array}$$

where $\ell_{i}\equiv \left(\ell_{ix},\ell_{iy},\ell_{iz}\right)$ and $S_{i}\equiv \left(S_{ix},S_{iy},S_{iz}\right)$ represent, respectively, continuously varying angular momenta and spin variables at each site of the linear chain, whereas $\sum_{\langle ij\rangle }$ denote summations over pairs of nearest-neighbor spins; herein, we set, without loss of generality, $k_{B}$ , moments of inertia, and ferromagnetic couplings, all equal to the unit. Moreover, spins present the unit norm, $S_{i}^{2}=1$ , and at each site, angular momentum $\ell_{i}$ must be perpendicular to $S_{i}$ , yielding $\ell_{i}\cdot S_{i}=0$ ; these two constraints are imposed at the initial state and should be preserved throughout the whole time evolution.

One should notice that, in contrast to a system of coupled classical XY rotators, where canonical conjugate polar coordinates are commonly used [45], in the Heisenberg case, one often chooses Cartesian coordinates [50,51,52]. The reason for this is essentially technical, since in terms of spherical coordinates (more precisely, θ,ϕ , and their canonical conjugates $\ell_{\theta },\ell_{\phi }$ ), a troublesome term $\left(1/\sin^{2}\theta \right)$ appears in the corresponding equations of motion, leading to numerical difficulties [53,54]. However, some of the analytical results to be derived next recover those of the classical inertial XY model for $S_{i}=\left(\sin\theta_{i},\cos\theta_{i},0\right)$ and $\ell_{i}=\ell_{i}\hat{z}$ .

It is important to mention that previous research on the thermal conductivity has been carried out for a classical one-dimensional Heisenberg spin model, by using Monte Carlo and Langevin numerical simulations [55], as well as for a classical one-dimensional spin-phonon system, through linear-response theory and the Green–Kubo formula [56]. These investigations did not take into account the kinetic contribution in Equation (9), so that in order to obtain the thermal conductivity they assumed the validity of Fourier’s law. The main advantage of the introduction of the kinetic term in Equation (9) concerns the possibility of deriving equations of motion, making it feasible to follow the time evolution of the system through molecular-dynamics simulations, by a numerical integration of such equations. This technique allows one to validate Fourier’s law, as well as obtain its thermal conductivity directly.

**Figure 1:**
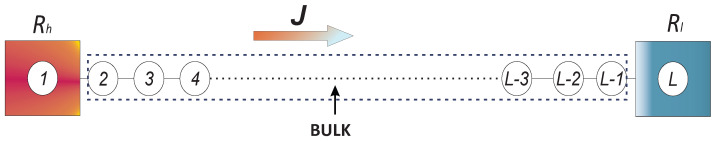
Illustration of the system defined in Equation (9), where the rotators at extremities of the chain are subjected to heat baths at different temperatures. The hot ( $R_{h}$ ) and cold ( $R_{l}$ ) reservoirs are at temperatures $T_{h}=T\left(1+\varepsilon \right)$ and $T_{l}=T\left(1-\varepsilon \right)$ , respectively, leading to an average heat flux J=Jx throughout the bulk (see text). The rotators at sites i=2,⋯,L−1 interact with their respective nearest neighbors.

In order to carry out this procedure, we consider an open chain of rotators with the first and last particles in thermal contact with heat baths at higher and lower temperatures, $T_{h}$ and $T_{l}$ ( $T_{h}>T_{l}$ ), respectively (cf. Figure 1), whereas all remaining rotators ( i=2,⋯,L−1 ) follow their usual equations of motion (see, e.g., Refs. [50,51,52]). In this way, one has for sites i=2,⋯,L−1 ,

(10) $$\begin{array}{c} \begin{array}{cc} \; & {\dot{S}}_{i}=\ell_{i}\times S_{i}\;,\\ \; & {\dot{\ell }}_{i}=S_{i}\times \left(S_{i+1}+S_{i-1}\right)\;, \end{array} \end{array}$$

whereas the rotators at extremities follow standard Langevin dynamics,

(11) $$\begin{array}{c} \begin{array}{cc} \; & {\dot{\ell }}_{1}=-\gamma_{h}\ell_{1}+S_{1}\times S_{2}+\eta_{h}\;,\\ \; & {\dot{\ell }}_{L}=-\gamma_{l}\ell_{L}+S_{L}\times S_{L-1}+\eta_{l}\;. \end{array} \end{array}$$

Above, $\gamma_{h}$ and $\gamma_{l}$ represent friction coefficients, whereas $\eta_{h}$ and $\eta_{l}$ denote independent three-dimensional vectors, $\eta_{h}\equiv \left(\eta_{hx},\eta_{hy},\eta_{hz}\right)$ , $\eta_{l}\equiv \left(\eta_{lx},\eta_{ly},\eta_{lz}\right)$ , where each Cartesian component stands for a Gaussian white noise with zero mean and correlated in time,

(12) $$\begin{array}{c} \begin{array}{cc} \; & \langle \eta_{h\mu }\left(t\right)\rangle =\langle \eta_{l\mu }\left(t\right)\rangle =0\;,\\ \; & \langle \eta_{h\mu }\left(t\right)\eta_{l\nu }\left(t^{\prime }\right)\rangle =\langle \eta_{h\mu }\left(t^{\prime }\right)\eta_{l\nu }\left(t\right)\rangle =0\;,\\ \; & \langle \eta_{h\mu }\left(t\right)\eta_{h\nu }\left(t^{\prime }\right)\rangle =2\delta_{\mu \nu }\gamma_{h}T_{h}\delta \left(t-t^{\prime }\right)\;,\\ \; & \langle \eta_{l\mu }\left(t\right)\eta_{l\nu }\left(t^{\prime }\right)\rangle =2\delta_{\mu \nu }\gamma_{l}T_{l}\delta \left(t-t^{\prime }\right)\;, \end{array} \end{array}$$

with the indexes μ and ν denoting Cartesian components; from now on, we will set the friction coefficients $\gamma_{h}$ and $\gamma_{l}$ equal to the unit. One should mention that different types of thermostats have been used to investigate transport properties in systems out of equilibrium (see, e.g., Ref. [42] for an application of Nosé–Hoover thermostats to a system of interacting planar rotators); however, for the present Heisenberg chain, we found it more convenient to use standard Langevin thermostats, as defined above.

The condition of a constant norm for the spin variables yields

(13) $$\begin{array}{c} \frac{dS_{i}}{dt}=\frac{d{\left(S_{i}\cdot S_{i}\right)}^{1/2}}{dt}=0\;\Rightarrow \;S_{i}\cdot {\dot{S}}_{i}=0\;,\; \end{array}$$

which should be used together with $\ell_{i}\cdot S_{i}=0$ in order to eliminate ${\ddot{\ell }}_{i}$ and calculate ${\ddot{S}}_{i}$ from Equations (10) and (11). For rotators at sites i=2,⋯,L−1 , one has

(14) $$\begin{array}{c} \begin{array}{cc} \; & {\ddot{S}}_{i}=\left(S_{i+1}+S_{i-1}\right)-\left[S_{i}\cdot \left(S_{i+1}+S_{i-1}\right)+{\dot{S}}_{i}^{2}\right]S_{i}\;, \end{array}\; \end{array}$$

whereas, for those at extremities,

(15) $$\begin{array}{c} \begin{array}{cc} \; & {\ddot{S}}_{1}=-{\dot{S}}_{1}+S_{2}-\left[S_{1}\cdot S_{2}+{\dot{S}}_{1}^{2}\right]S_{1}+S_{1}\times \eta_{h}\;,\\ \; & {\ddot{S}}_{L}=-{\dot{S}}_{L}+S_{L-1}-\left[S_{L}\cdot S_{L-1}+{\dot{S}}_{L}^{2}\right]S_{L}+S_{L}\times \eta_{l}\;. \end{array}\; \end{array}$$

For the system illustrated in Figure 1, we will consider the temperatures of the heat baths differing by 2ε , with ε representing a positive dimensionless parameter; moreover, the temperature parameter $T=(T_{h}+T_{l})/2$ will vary in a certain range of positive values. Equations (14) and (15) are transformed into first-order differential equations (e.g., by defining a new variable $V_{i}\equiv {\dot{S}}_{i}$ ) to be solved numerically through the velocity Verlet method [57,58], with a time step dt=0.005 , for different lattice sizes *L* (please, see the Appendix A). The rotators at the bulk (i =2,⋯,L−1 ) follow a continuity equation,

(16) $$\begin{array}{c} \frac{dE_{i}}{dt}=-\left(J_{i}-J_{i-1}\right)\;,\; \end{array}$$

where

(17) $$\begin{array}{c} E_{i}=\frac{1}{2}\;\ell_{i}^{2}+\frac{1}{2}\sum_{j=i\pm 1}\left(1-S_{i}\cdot S_{j}\right)\;,\; \end{array}$$

so the stationary state is attained for $(dE_{i}/dt)=0$ , i.e., $J_{i}=J_{i-1}$ . The derivation is simple, since from Equation (13) and $\ell_{i}\cdot S_{i}=0$ , we have ${\dot{S}}_{i}^{2}=\ell_{i}^{2}$ , hence,

(18) $$\frac{d}{dt}E_{i}={\dot{S}}_{i}\cdot {\ddot{S}}_{i}-\frac{1}{2}\left[{\dot{S}}_{i}\cdot \left(S_{i+1}+S_{i-1}\right)+S_{i}\cdot \left({\dot{S}}_{i+1}+{\dot{S}}_{i-1}\right)\right]\;.$$

This equation, together with Equation (14), yields

(19) $$\frac{d}{dt}E_{i}=\frac{1}{2}\left[{\dot{S}}_{i}\cdot \left(S_{i+1}+S_{i-1}\right)-S_{i}\cdot \left({\dot{S}}_{i+1}+{\dot{S}}_{i-1}\right)\right]=0$$

at the stationary state. Data are obtained at stationary states, which, as usual, take longer to reach for increasing lattice sizes. For numerical reasons, to decrease fluctuations in the bulk due to the noise, we compute an average heat flux by discarding a certain number of particles *p* near the extremities (typically p≃0.15L ). In this way, we define an average heat flux as

(20) $$\begin{array}{c} J\equiv \frac{1}{L-2p}\sum_{i=p+1}^{L-p}\langle J_{i}\rangle \;,\; \end{array}$$

(21) $$\begin{array}{c} J_{i}=\frac{1}{2}\left(S_{i}\cdot {\dot{S}}_{i+1}-S_{i+1}\cdot {\dot{S}}_{i}\right)\;,\; \end{array}$$

whereas 〈..〉 denotes time and sample averages, which will be described next.

Let us emphasize that for $S_{i}=\left(\sin\theta_{i},\cos\theta_{i},0\right)$ and $\ell_{i}=\ell_{i}\hat{z}$ , one recovers the expression for the heat flux of the classical inertial XY model, i.e., $J_{i}=\frac{1}{2}\left(\ell_{i}+\ell_{i+1}\right)\sin\left(\theta_{i}-\theta_{i+1}\right)$ [45,59], showing the appropriateness of the Cartesian-coordinate approach used herein for the classical inertial Heisenberg model.

Let us now describe the time evolution procedure; for a time step dt=0.005 , each unit of time corresponds to 200 integrations of the equations of motion. We considered a transient of $5\times 10^{7}$ time units to compute the averages $\langle J_{i}\rangle$ in Equation (20), and checked that this transient time was sufficient to fulfill the condition $J_{i}=J_{i-1}$ (within, at least, a three-decimal digits accuracy), for all values of *L* analyzed. After that, simulations were carried out for an additional interval of $2\times 10^{8}$ time units (leading to a total time of $2.5\times 10^{8}$ for each simulation). The interval $2\times 10^{8}$ was divided into 80 equally spaced windows of $2.5\times 10^{6}$ time units, so that time averages were taken inside each window; then an additional sample average was taken over these 80 time windows, leading to the averages $\langle J_{i}\rangle$ .

Using the results of Equation (20), one may calculate the thermal conductivity of Equation (1), and consequently, the thermal conductance,

(22) $$\begin{array}{c} \sigma =\frac{J}{T_{h}-T_{l}}=\frac{J}{2T\varepsilon }\equiv \frac{\kappa }{L}\;.\; \end{array}$$

In the next section, we present the results of both quantities, obtained from the numerical procedure described above.

## 3. Results

We simulate the system of Figure 1 for different lattice sizes, namely, L=50, 70, 100, 140, considering the heat-bath temperatures differing by 2ε , with ε=0.125 . The temperature parameter $T=(T_{h}+T_{l})/2$ varied in the interval 0<T≤3.5 , capturing both low- and high-temperature regimes. The values of *L* ( L≥50 ) were chosen adequately to guarantee that the thermal conductivity κ did not present any dependence on the size *L* in the high-temperature regime, as expected.

**Figure 2:**
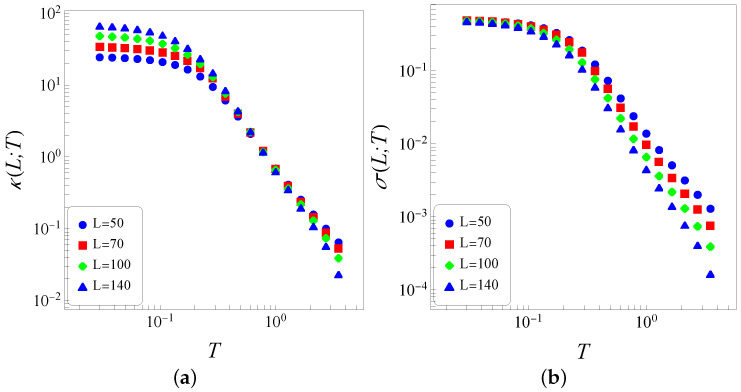
(Color online) Numerical data for the thermal conductivity [panel (**a**)] and thermal conductance [panel (**b**)] are represented versus temperature (log–log plots) for different sizes ( L=50,70,100,140 ) of the one-dimensional classical inertial Heisenberg model. One notices a crossover between the low- and high-temperature regimes for T≃0.3 . As expected, higher temperatures amplify the effects of the multiplicative noise, which is proportional to the square root of the corresponding temperatures ( $T_{h},T_{l}$ ), currently leading to larger fluctuations in numerical data, as shown in panel (**a**). All quantities shown are dimensionless.

In Figure 2, we present numerical data for the thermal conductivity Figure 2a and thermal conductance Figure 2b versus temperature (log–log representations) and different sizes *L*. The similar qualitative behaviors of the data displayed in both properties of Figure 2, for different values of *L*, evidence that the sizes considered in the present analysis ( L≥50 ) are sufficiently large, in the sense that finite-size effects do not play a relevant role. In Figure 2a, we exhibit κ(L,T) (the dependence of the thermal conductivity on the size *L*, used herein, will become clear below), showing a crossover between two distinct regimes (for T≃0.3 ), as described next. (i) A low-temperature regime, where κ depends on the size *L*, decreasing smoothly for increasing temperatures (*L* fixed). The plots of Figure 2a show that, in the limit T→0 , an extrapolated value, $\kappa \left(L,0\right)\equiv \lim_{T\to 0}\kappa \left(L,T\right)$ , increases with *L*. Such a low-temperature increase with *L* has been observed in other one-dimensional models (see, e.g., Refs. [42,43,44,45]) and is reminiscent of the behavior expected for a chain of coupled classical harmonic oscillators. This anomaly is attributed to the classical approach used herein, indicating that for low temperatures, a quantum–mechanical procedure should be applied. (ii) A high-temperature regime, where κ essentially does not depend on *L* (in the limit L→∞ ), as expected from Fourier’s law. Moreover, in this regime, one notices that κ decreases with the temperature as it generally occurs with liquids and solids. For increasing temperature, the thermal conductivity of most liquids usually decreases as the liquid expands and the molecules move apart; in the case of solids, due to lattice distortions, higher temperatures make it more difficult for electrons to flow, leading to a reduction in their thermal conductivity. The results of Figure 2a indicate that the thermal conductivity becomes independent of the lattice size in the limit L→∞ , scaling with the temperature as $\kappa \left(T\right)∼T^{-2.25}$ at high temperatures. Therefore, the system becomes a thermal insulator at high temperatures, approaching this state according to $\kappa \left(T\right)∼T^{-2.25}$ . Despite the simplicity of the one-dimensional classical inertial Heisenberg model of Figure 1, the present results are very close to experimental verifications in some antiferromagnetic electrical insulators, such as the Heisenberg chain cuprates ${\mathrm{Sr}}_{2}{\mathrm{CuO}}_{3}$ and ${\mathrm{SrCuO}}_{2}$ , for which the thermal conductivity is well-fitted by a $1/T^{2}$ law at high temperatures [4]. We should note that the one-dimensional Heisenberg model with nearest-neighbor ferromagnetic interactions, defined by the Hamiltonian of Equation (9), does not present an equilibrium phase transition, being characterized by a paramagnetic state for all temperatures T>0 . In this case, one may perform the following transformations in the Hamiltonian of Equation (9), leaving it unaltered: 1/2→−1/2 (which incorporates the coupling constant), as well as $S_{j}\to -S_{j}$ , keeping $S_{i}$ unchanged. Consequently, the Hamiltonian of Equation (9) applies to antiferromagnetic systems at high temperatures, as well. 0

The same data of Figure 2a are exhibited in Figure 2b where we plot the thermal conductance σ(L,T)=κ(L,T)/L versus temperature, characterized by the two distinct temperature regimes described above. The low-temperature regime shows that the zero-temperature extrapolated value κ(L,0) scales as κ(L,0)∼L , leading to $\sigma \left(L,0\right)\equiv \lim_{T\to 0}\kappa \left(L,T\right)/L≃0.5$ . Such low-temperature results are in full agreement with those obtained in previous simulations of coupled classical XY rotators [42,43,44,45]. On the other hand, in the high-temperature regime, the thermal conductance presents a dependence on *L*, as expected.

**Figure 3:**
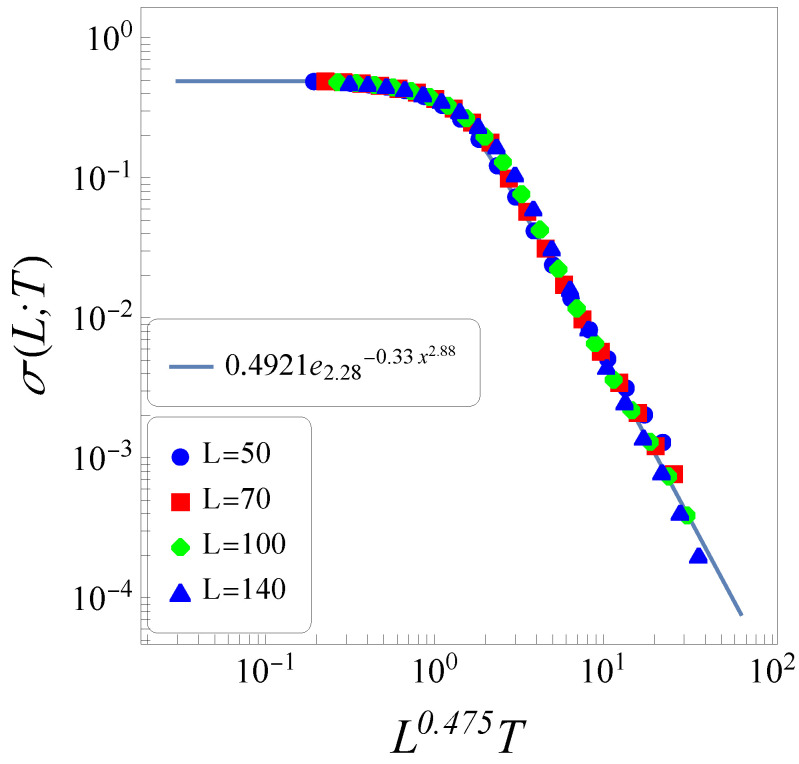
The plots for the thermal conductance of Figure 2b are shown in a log–log representation, for a conveniently chosen abscissa ( $x=L^{0.475}T$ ), leading to a collapse of data for all values of *L* considered. The fitting (full line) is given by the function of Equation (23).

In Figure 3, we exhibit the thermal-conductance data of Figure 2b in conveniently chosen variables, yielding a data collapse for all values of *L* considered. The full line essentially represents the form of Equation (8), so that one writes

(23) $$\sigma \left(L,T\right)=A\exp_{q}\left(-Bx^{\eta }\right)\;,\;$$

where $x=L^{0.475}T$ , q=2.28±0.04 , η=2.88±0.04 , A=0.492±0.002 , and B=0.33±0.04 . Notice that this value of η lies outside the range of what is commonly known as “stretched” [cf. Equation (8)], so that the form above should be considered rather as a “shrinked” *q*-exponential.

It should be mentioned that, in the case of coupled nearest-neighbor-interacting classical XY rotators on *d*-dimensional lattices ( d=1,2,3 ) [45], the thermal conductance was also fitted by the form of Equation (23), with values of η(d)>2 . In particular, in the one-dimensional case, such a fitting was attained for $x=L^{0.3}T$ , q=1.7 , and η=2.335 , showing that these numbers present a dependence on the number of spin components ( n=2 , for XY spins and n=3 , for Heisenberg spins), as well as on the lattice dimension *d*. It is important to mention that the generalized forms in Equations (8) and (23) have been used in the literature for an appropriate description of a wide variety of physical phenomena, like velocity measurements in a turbulent Couette–Taylor flow [60], relaxation curves of RKKY spin glasses, such as CuMn and AuFe [61], cumulative distribution for the magnitude of earthquakes [62], and more recently, for the thermal conductance of a system of interacting XY rotators [45]. Moreover, its associated entropic form has been studied in detail in Ref. [49].

By defining the abscissa variable of Figure 3 in the general form $x=L^{\gamma (n,d)}T$ , and using the *q*-exponential definition of Equation (3), the slope of the high-temperature part of the thermal-conductance data scales with *L*, as

(24) $$\sigma ∼L^{-[\eta (n,d)\gamma (n,d)]/[q(n,d)-1]}\;,\;$$

where we introduce the dependence (n,d) on all indices. Since the thermal conductivity ( κ=Lσ ) should not depend on the size *L* (in the limit L→∞ ), Fourier’s law becomes valid for

(25) $$\frac{\eta (n,d)\gamma (n,d)}{q(n,d)-1}=1\;.\;$$

The data of Figure 3 lead to [η(3,1)γ(3,1)]/[q(3,1)−1]=1.069±0.083 , whereas those for XY rotators on *d*-dimensional lattices yield 1.0007,0.95 , and 0.93 , for d=1,2 , and 3, respectively [45], indicating the validation of Fourier’s law for systems of coupled nearest-neighbor-interacting classical *n*-vector rotators, through the thermal conductance form of Equation (23).

Recently, similar analyses were carried out for an XY Hamiltonian with anisotropies, in such a way to approach the Ising model in particular limits [63]. All the results for the quantity in Equation (25), computed up to the moment, are summarized in Table 1, where one notices that finite-size effects play an important role in increasing dimensions, as expected.

## 4. Conclusions

We studied the heat flow along a one-dimensional classical inertial Heisenberg model of linear size *L*, by considering the first and last particles in thermal contact with heat baths at different temperatures, $T_{h}$ and $T_{l}$ ( $T_{h}>T_{l}$ ), respectively. These particles at the extremities of the chain were subjected to standard Langevin dynamics, whereas all remaining rotators ( i=2,⋯,L−1 ) interacted by means of nearest-neighbor ferromagnetic couplings and evolved in time following their own classical equations of motion, being investigated numerically through molecular-dynamics numerical simulations.

Fourier’s law for the heat flux was verified numerically, and both thermal conductivity κ(T) and thermal conductance σ(L,T)=κ(T)/L were computed, by defining $T=(T_{h}+T_{l})/2$ . The slope of the high-temperature part of thermal-conductance data scales with the system size was $\sigma ∼L^{-1.069}$ , indicating that in the limit L→∞ , one should obtain a thermal conductivity independent of *L*. Indeed, in this limit, we found $\kappa \left(T\right)∼T^{-2.25}$ for high temperatures. The thermal-conductance data were well-fitted by the function $\sigma \left(L,T\right)=A\exp_{q}\left(-Bx^{\eta }\right)$ , typical of nonextensive statistical mechanics, where *A* and *B* are constants, $x=L^{0.475}T$ , q=2.28±0.04 , and η=2.88±0.04 . This fitting augments the applicability of such a function, which has been used for describing several physical phenomena in the literature, like velocity measurements in a turbulent Couette–Taylor flow [60], relaxation curves of RKKY spin glasses [61], cumulative distribution for the magnitude of earthquakes [62], and thermal conductance of a system of interacting XY rotators [45]. Since the value of η found herein lies outside the range of what is commonly known as “stretched” (0<η≤1) , herein, we refer to this fitting function of a “shrinked” *q*-exponential. The present results reinforce those obtained recently for XY rotators on *d*-dimensional lattices [45], indicating that Fourier’s law should be generally valid for systems of coupled nearest-neighbor-interacting classical *n*-vector rotators, through the “shrinked” *q*-exponential function for the thermal conductance, with the indices q(n,d) and η(n,d) presenting a dependence on the number of spin components and lattice dimension.

Despite the simplicity of the model considered herein, the results for the thermal thermal conductivity at high temperatures ( $\kappa \left(T\right)∼T^{-2.25}$ ) are very close to experimental verifications in some antiferromagnetic electrical insulators, such as the Heisenberg chain cuprates ${\mathrm{Sr}}_{2}{\mathrm{CuO}}_{3}$ and ${\mathrm{SrCuO}}_{2}$ , for which thermal conductivity is well-fitted by a $1/T^{2}$ law at high temperatures [4]. At equilibrium, the present model exhibits a paramagnetic state for all temperatures, so that its Hamiltonian may be shown to cover both ferromagnetic and antiferromagnetic systems. The present results show that even for models exhibiting simple equilibrium properties, one may have out-of-equilibrium regimes characterized by transport properties typical of nonextensive statistical mechanics, like the ones found herein. Since nonextensive statistical mechanics have been used in the description of a wide variety of complex systems, one expects that the present results should be applicable to many of these systems in diverse, non-equilibrium regimes.

In summary, we demonstrated that (i) for the classical one-dimensional inertial ferromagnetic Heisenberg model, the (macroscopic) Fourier-law is validated from (microscopic) first principles, i.e., the temperature-dependent thermal conductivity is, in the high-temperature regime, finite and independent of the system size (the low-temperature regime is to be handled within a quantum grounding, which is out of the goal of the present paper); (ii) For all temperatures and sizes, the thermal conductivity appears to be consistent with *q*-statistics since it can be neatly collapsed within a shrunken *q*-exponential form; (iii) within this shrunken *q*-exponential form, a single universal condition, namely $\frac{\eta (n,d)\gamma (n,d)}{q(n,d)-1}=1$ , validates the Fourier law for the *n*-vector models for n=1,2,3 , which constitutes a numerical indication that this centennial macroscopic law is possibly valid for all values of (n,d) , where n→∞ and n→0 correspond to the spherical model and ‘self-avoiding walk’, respectively. It is not our present aim to review the rich existing literature on the validity of the Fourier law within diverse classical and quantum approaches, but we rather restrict our focus to analytical and numerical first-principle approaches of classical systems that are similar to the present one.

## Figures and Tables

**Table 1:** Values of the ratio ηγ/(q−1) (highlighted in blue color) analyzed up to the moment: n=1 ( d=1 ) [63], n=2 (dimensions d=1,2,3 ) [45], together with the present results for n=3 ( d=1 ). In all cases studied, the limit of Equation (25) is numerically approached.

$\frac{\eta \gamma }{q-1}$	d=1 (linear chain)	d=2 (square lattice)	d=3 (simple cubic lattice)
n=1 (Ising ferromagnet)	1.0063 q=1.65,η=1.94,γ=0.336	-	-
n=2 ( XY ferromagnet)	1.0007 q=1.7,η=2.335,γ=0.3	0.95 q=3.2,η=5.23,γ=0.4	0.93 q=3.5,η=5.42,γ=0.43
n=3 (Heisenberg ferromagnet)	1.069 q=2.28,η=2.88,γ=0.475	-	-

## Data Availability

All data will be available upon request.

## Appendix A. Numerical Procedures

Let us focus on the numerical integration of the equations of motion of the classical inertial Heisenberg chain. Considering the change of variable ${\dot{S}}_{i}=V_{i}$ , we have the following equations of motion:

(A1)
$$\begin{array}{c} \begin{array}{cc} \; & {\dot{S}}_{i}=V_{i}\;\;\mathrm{for}\;\mathrm{all}\;i\\ \; & {\dot{V}}_{i}=\left(S_{i+1}+S_{i-1}\right)-\left[S_{i}\cdot \left(S_{i+1}+S_{i-1}\right)+V_{i}^{2}\right]S_{i}\;,\;\;i=2,\ldots ,L-1\\ \; & {\dot{V}}_{1}=-V_{1}+S_{2}-\left[S_{1}\cdot S_{2}+V_{1}^{2}\right]S_{1}+S_{1}\times \eta_{h}\;,\\ \; & {\dot{V}}_{L}=-V_{L}+S_{L-1}-\left[S_{L}\cdot S_{L-1}+V_{L}^{2}\right]S_{L}+S_{L}\times \eta_{l}\;. \end{array}\; \end{array}$$

which is a system of 6L first-order differential equations. To solve the set of equations entirely with the velocity Verlet method, we need to define $\eta_{h/l}=\sqrt{2T_{h/l}/dt}\;w_{h/l}$ , where *w* is a vector of dimensionless Gaussian white noises and dt is the time-step of the integration. Therefore, we have the following discretized procedure:

(A2)
$$\begin{array}{c} \begin{array}{cc} \; & S_{i}^{k+1}=S_{i}^{k}+V_{i}\;dt+\frac{1}{2}F_{i}^{k}\;{\left(dt\right)}^{2}\\ \; & V_{i}^{k+1}=V_{i}^{k}+\frac{1}{2}\left(F_{i}^{k}+F_{i}^{k+1}\right)dt \end{array} \end{array}$$

where the generalized forces are as follows:

(A3)
$$\begin{array}{c} \begin{array}{cc} \; & F_{i}=\left(S_{i+1}+S_{i-1}\right)-\left[S_{i}\cdot \left(S_{i+1}+S_{i-1}\right)+V_{i}^{2}\right]S_{i}\;,\;\;i=2,\ldots ,L-1\\ \; & F_{1}=-V_{1}+S_{2}-\left[S_{1}\cdot S_{2}+V_{1}^{2}\right]S_{1}+\sqrt{\frac{2T_{h}}{dt}}\left(S_{1}\times w_{h}\right)\;,\\ \; & F_{L}=-V_{L}+S_{L-1}-\left[S_{L}\cdot S_{L-1}+V_{L}^{2}\right]S_{L}+\sqrt{\frac{2T_{l}}{dt}}\left(S_{L}\times w_{l}\right)\;. \end{array}\; \end{array}$$

Notice that ${\left(dt\right)}^{-1/2}$ in the stochastic part of the forces is equivalent to ${\left(dt\right)}^{3/2}$ and ${\left(dt\right)}^{1/2}$ in $F_{i}^{k}\;{\left(dt\right)}^{2}$ and $F_{i}^{k}\;dt$ respectively; this equivalence contains weak order 1/2 and strong order 3/2 properties.
